# Integrin αV modulates the cellular pharmacology of copper and cisplatin by regulating expression of the influx transporter CTR1

**DOI:** 10.18632/oncoscience.22

**Published:** 2014-03-24

**Authors:** Xinjian Lin, Xiying Shang, Gerald Manorek, Mariama Fofana, Howell Stephen B.

**Affiliations:** ^1^ Department of Medicine and UC San Diego Moores Cancer Center, University of California, San Diego, La Jolla, CA

**Keywords:** Integrin αV, copper, cisplatin, CTR1, Sp1, cellular pharmacology

## Abstract

The αV integrin is expressed in most cancer cells where it regulates a diverse array of cellular functions essential to the initiation, progression and metastasis of solid tumors. However, little is known about how αV integrin modulates cellular sensitivity to chemotherapeutic agents, particularly the platinum drugs. In this study, we found that down-regulation of αV sensitized human M21 cells to cisplatin (cDDP) through up-regulation of the copper influx transporter CTR1. Cells selected for low αV integrin expression (M21L) were more sensitive to cDDP, accompanied by increase in CTR1 mRNA and CTR1 protein levels, more intracellular cDDP accumulation and cDDP DNA adduct formation. Basal copper (Cu) content, Cu uptake, and Cu cytotoxicity were also increased. Transfection of a luciferase reporter construct containing the *hCTR1* promoter sequence revealed an increase of the *hCTR1* transcription activity in M21L cells. The basis for the increased hCTR1 transcription was related to an increase in the steady-state level of Sp1, a transcription factor known to drive *hCTR1* expression. These results indicate that the αV integrin modulates sensitivity of human cells to the cytotoxic effect of cDDP by regulating expression of the Cu transporter CTR1, and introduce the concept that αV expression is linked to Cu homeostasis.

## INTRODUCTION

Integrins are cell-surface glycoprotein receptors composed of a set of non-covalently associated α and β subunits. There are 18 α and 8 β subunits capable of forming 24 known combinations that preferentially bind to distinct ECM proteins and account for the structural and functional diversity of the integrin family. Studies correlating integrin expression in human tumors with clinical outcomes such as survival and metastasis have identified several integrins that appear to have an important roles in cancer progression including α_v_β_3_, α_v_β_5_, α_5_β_1_, α_6_β_4_, α_4_β_1_ and α_v_β_6_[1]. There is also evidence that integrins may influence the sensitivity of cancers to chemotherapeutic agents since increased expression of some integrin subunits and/or heterodimers has been found in drug resistant cells [2]. Drug resistance induced by integrin-mediated interaction of tumor cells with the interstitial or extracellular matrix (ECM) has been reported in a variety of hematological malignancies [3-6] and solid cancers [7-9], and has been identified as “cell adhesion-mediated drug resistance”. Integrins may also modulate the threshold for the triggering of apoptosis following treatment with chemotherapeutic drugs. For instance, β1-integrin mediates resistance of leukemia cells to the apoptotic effects of chemotherapeutic drugs through regulation of the expression of Bcl-2 family proteins [6, 10]. Moreover, the αV subunit, which is expressed in most cancer cells and plays an essential role in the formation of both cell–matrix and cell–cell interactions [11, 12], influences multiple processes including proliferation, survival and apoptosis [13-16].

Cisplatin (cDDP) is an effective first-line therapy for many types of cancer but the rapid development of resistance during therapy remains a major clinical challenge. cDDP is thought to kill cells predominantly by forming adducts in DNA that block transcription and DNA replication. Mechanisms implicated in cellular resistance include reduced drug uptake, increased drug efflux, increased DNA repair, increased tolerance of DNA damage, and an aberrations in apoptosis pathways (reviewed in [17, 18]). More recently, the copper (Cu) transporters have been found to modulate the cellular pharmacology of the platinum (Pt) drugs [19-21]. Resistance appears to be multi-factorial in origin with no single overarching mechanism predominating even within the same histological type of tumor. Novel insights into molecular mechanisms of resistance are important to the goal of identifying patients whose tumors have a high probability of responding to cDDP, and avoiding administration of this drug to patients unlikely to benefit from treatment.

We have observed that the expression of the αV integrin subunit influences sensitivity to cDDP. In this study we used the human melanoma cell line M21 that expresses wild-type αV, and its stable variant M21L that lacks αV gene expression, to investigate the mechanism of this effect [22, 23]. We report here that loss of αV integrin renders cells hypersensitive to the cytotoxic effect of cDDP, which is mediated through the Sp1 transcription factor that transcriptionally up-regulates the expression of Cu influx transporter CTR1 leading to both enhanced cDDP uptake, adduct formation and cell kill and changes in the level of intracellular Cu.

## RESULTS

### αV integrin controls sensitivity to cDDP

**Figure 1:**
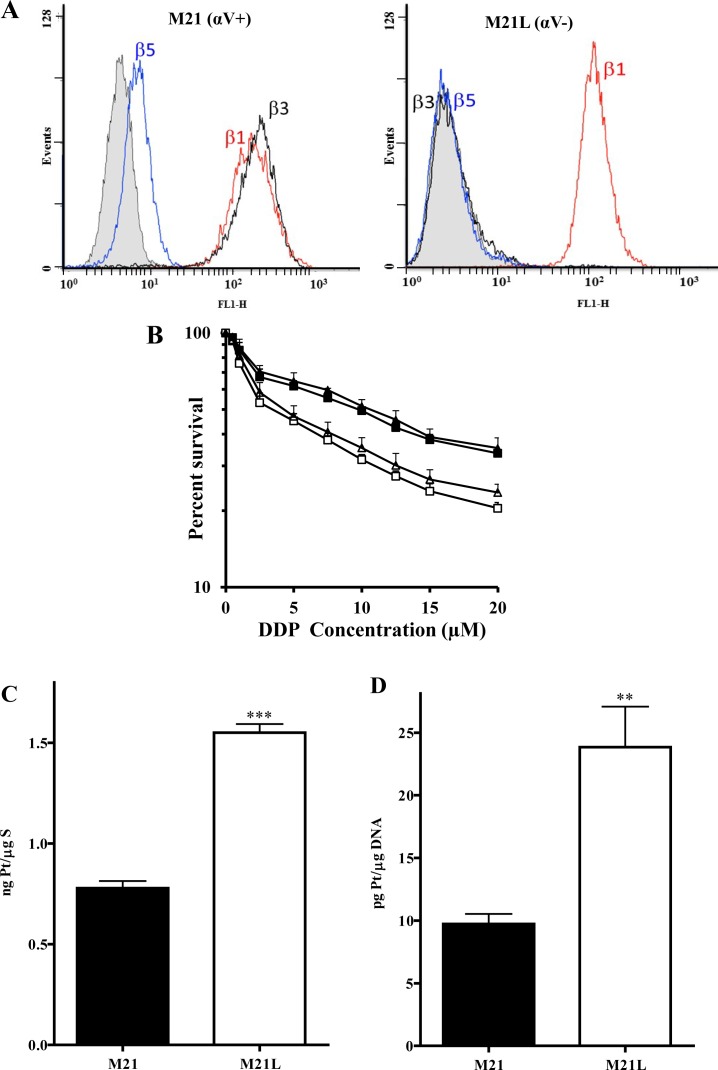
Effect of loss of αV integrin on cDDP sensitivity, uptake and DNA adduct formation (A) Flow cytometric analysis of αVβ3, αVβ5 and β1 expression on M21 and M21L cells using anti-αVβ3, anti-αVβ5 or anti-β1 antibodies. The gray filled area depicts cells stained with the second antibody only which serves as a negative control. (B) *In vitro* cytotoxicity of cDDP to the parental M21 (▲), αV integrin-depleted M21L (Δ), αV cDNA-transfected M21L4 (■) and empty vector-transfected M21L12 (□) cells. The cells were treated with increasing concentrations of cDDP for 96 h and CCK-8 assay was used to quantify cell viability. Each data point represents the mean of 3 independent experiments each performed with triplicate cultures. (C) Whole cell Pt accumulation in the cells treated with 30 μM cDDP for 1 h. (D) DNA-Pt adduct levels following the same exposure. Vertical bars, ± SEM. ***, p<0.001 vs M21 control cells; **, p<0.01 vs M21 control cells.

To determine whether the αV integrin subunit is a regulator of cDDP sensitivity, the cytotoxicity of cDDP to the parental M21 line was compared to that for the M21L subline that had previously been selected for reduced αV expression [22, 23]. Fig.1A shows a flow cytometric analysis of the M21 and M21L cells that documents the absence of αV expression in the M21L cells as evidenced by the lack of detectable levels of both αVβ3 and αVβ5 expression. Both M21 and M21L expressed equivalent levels of β1 integrins.

As shown in Fig. 1B, loss of αV function increased cDDP sensitivity; the cDDP IC_50_ (mean ± SEM) for the parental M21 cells was 9.9 ± 0.7 μM whereas it was only 3.5 ± 0.9 μM for the αV integrin-negative M21L cells (p = 0.004). Therefore, reducing the expression of αV rendered M21L cells 2.8-fold hypersensitive to cDDP. To confirm a direct role for αV expression in controlling sensitivity, the effect of re-expressing αV in the M21L cells on the cDDP IC_50_ was determined. The M21L4 and M21L12 cells were previously produced by stably transfecting the M21L cells with either a full-length αV cDNA-containing vector to produce the M21L4 cells, or an empty vector control to produce the M21L12 cells [22, 23]. Intriguingly, re-expression of αV in the M21L4 cells significantly reduced their sensitivity to cDDP as compared to the empty vector-transfected M21L12 cells (IC_50_ 11.60 ± 1.21 versus 4.27 ± 0.62 μM, p = 0.008), confirming that expression of the αV integrin subunit controls sensitivity to cDDP in these cells.

### Loss of αV integrin increases cDDP accumulation

To determine whether the changes in sensitivity to cDDP resulted from differences in cellular cDDP accumulation, whole cell Pt content was measured by ICP-MS following a 1 h exposure to 30 μM cDDP. As shown in Fig. 1C, the parental M21 cells accumulated 0.77 ± 0.06 ng Pt/μg S whereas the M21L cells accumulated 1.55 ± 0.07 ng Pt/μg S, representing a 2.0-fold (p = 0.0002) increase in whole cell accumulation of this Pt-containing drug. Thus, at least part of the observed increase in drug sensitivity can be accounted for by an increase in drug accumulation. Formation of DNA-Pt adducts is believed to be the primary mechanism by which Pt-containing drugs cause cell death. Pt was measured in DNA isolated from the M21 and M21L cells exposed for 1 h to 30 μM cDDP (Fig. 1D). The αV integrin-negative M21L cells contained 23.9 ± 9.1 pg Pt/μg DNA, a 2.5-fold increase (p = 0.0029) relative to level of 9.7 ± 2.3 pg Pt/μg DNA in the parental M21 cells. Thus, increased whole cell accumulation was accompanied by a proportional increase in the amount of cDDP reacting with DNA.

### Loss of αV integrin increases the level of CTR1

**Figure 2:**
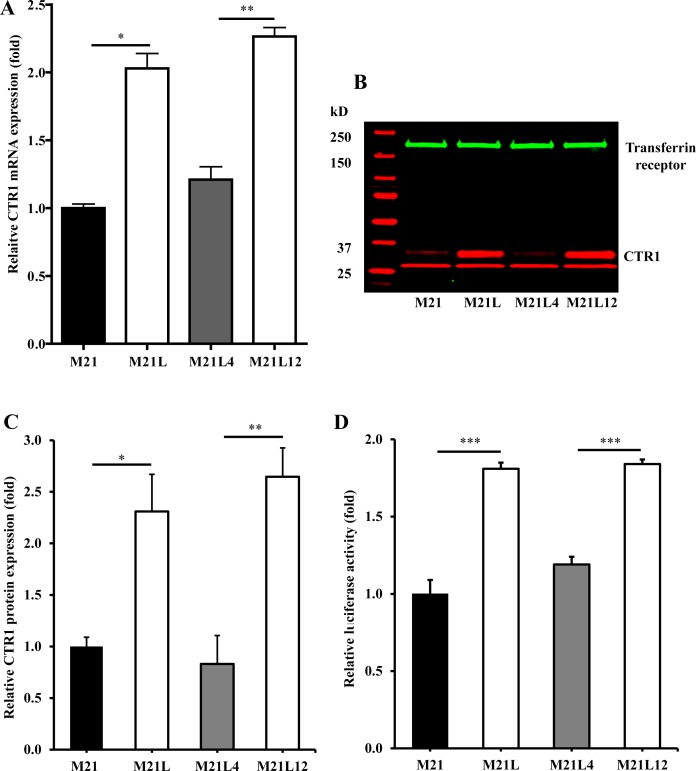
Loss of αV integrin increases the expression of CTR1 (A) qRT-PCR analysis of the effect of αV integrin on mRNA expression of CTR1. (B + C) Western blot analysis of the effect of αV integrin loss on CTR1 protein level. (D) Transcriptional regulation of CTR1 due to loss of αV integrin. The −227 to +303 genomic region containing the predicted CTR1 promoter region [24] was cloned into pGL3 basic luciferase vector and transfected into M21, M21L, M21L4 and M21L12 cells. The level of promoter activity was evaluated 48 h after transfection by measuring the luciferase activity normalized for variations in transfection efficiency and expressed as a fold change relative to the M21 control cells. Vertical bars, ± SEM, n=6. *, p<0.05 vs M21 control cells; **, p<0.01 vs M21L4 cells. ***p<0.001.

Given the observation that depletion of av integrin sensitized cells to cDDP, and that sensitivity to cDDP is mediated in part by the major Cu influx transporter CTR1, the relative expression of CTR1 was quantified by qRT-PCR and Western blot analysis. As shown in Fig. 2A, the mRNA level of CTR1 was 2.0 ± 0.19 - fold higher (p = 0.011) in the M21L cells as compared to that in the αV integrin-expressing wild-type M21 cells. Re-expression of the αV integrin by transfection of the M21L cells to produce the M21L4 cells reduced the CTR1 mRNA level toward that of the M21 cells whereas transfection of an empty vector to produce the M12L12 cells had no effect. Consistent with the change in mRNA level, the level of CTR1 protein was 2.3 ± 0.6-fold higher (p = 0.015) in the M21L cells than in M21 cells when assessed by Western blot (Fig. 2B and C). Re-expression of αV integrin in the M21L4 cells also decreased CTR1 protein levels whereas the empty vector-transfected M21L12 cells maintained a high level of CTR1 similar to that in the M21L cells. To further confirm that transcriptional mechanisms are involved in the regulation of CTR1, we transfected all 4 cells with a luciferase reporter vector containing the CTR1 promoter region from −227 to +330 [24] to evaluate changes in CTR1 promoter activity associated with the loss of αV integrin. As shown in Fig. 2D, transfection with this CTR1 promoter-dependent reporter plasmid demonstrated a 1.8-fold increase of reporter gene activity in the αV-negative M21L cells relative to that in the αV integrin wild-type M21 cells (p<0.001). Similarly, a significant reduction in the reporter activity was observed in the αV re-expressing M21L4 cells as compared with the empty vector-transfected M21L12 controls. These results suggest that a major component of the effect of loss of αV on CTR1 expression is at the transcriptional level.

**Figure 3:**
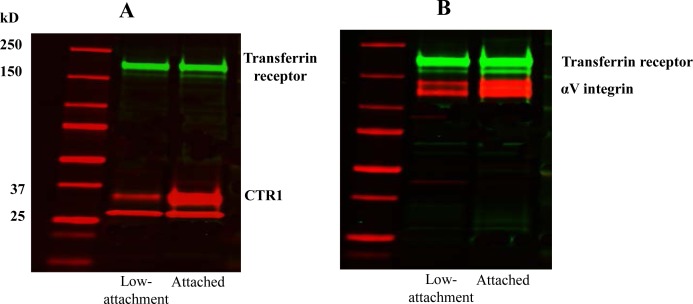
Effect of preventing M21 cell attachment on CTR1 and αV expression Cells grown on regular tissue culture plate or low attachment plate were harvested and subjected to Western blot analysis for detection of CTR1 (A) and αV (B) using the respective antibody.

Loss of αV reduces cell attachment so that the M21L cells grow partially in suspension [25]. To exclude the possibility that the increase in CTR1 levels observed in the M21L cells was due to the fact that they are not completely attached, the adherent M21 cells were forced to grow in suspension by seeding them on a low attachment plate and their expression of CTR1 was compared with M21 cells grown on regular tissue culture plate. Surprisingly, a marked decrease rather than increase of CTR1 expression was observed by Western blot analysis when the M21 cells were forced to grow unattached (Fig. 3A). The same was true for αV expression (Fig. 3B). It has been reported that integrin-associated cell-matrix engagement can disrupt adherens junctions by down-regulation of E-cadherin [26]. However, E-cadherin was not detectable in M21 cells when grown attached and growth on low attachment plate did not up-regulate E-cadherin (data not shown). Thus, the inability of M21L cells to attach is not the cause of the increase in CTR1 expression levels observed in these cells.

### Loss of αV integrin perturbs Cu homeostasis

**Figure 4:**
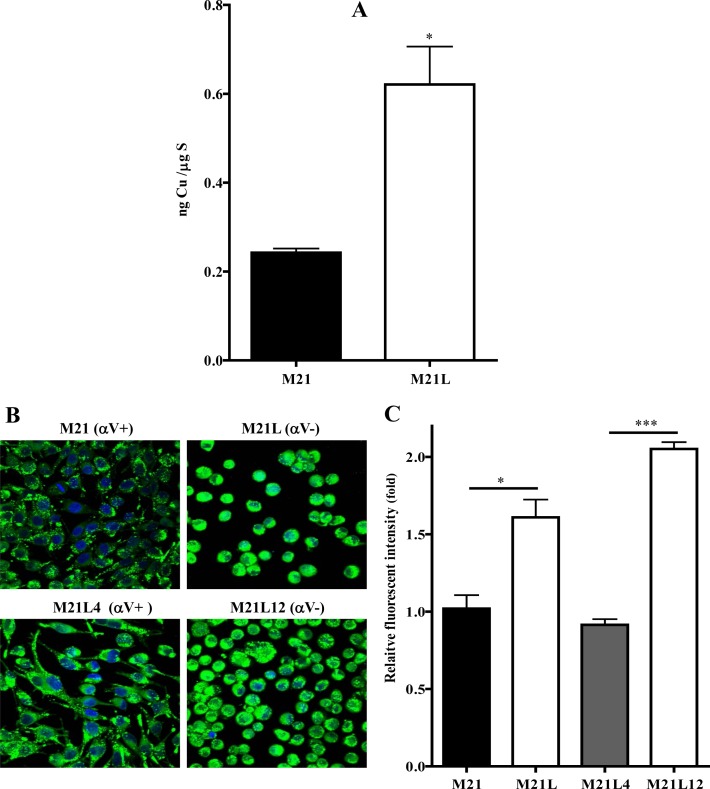
Effect of the loss of αV integrin on the basal Cu levels (A) Steady-state basal level of Cu in M21 and M21L cells measured by ICP-MS. (B) Molecular imaging of endogenous basal Cu in the cells with CS3. (C) Quantification of CS3 fluorescence by image J software. Vertical bars, ± SEM. *, p<0.05 vs M21 control cells; ***, p<0.001 vs M21L4.

CTR1 is the main Cu influx transporter in human cells and its down-regulation is associated with reduced accumulation of Cu and resistance to its cytotoxic effect [27]. To determine whether the change in CTR1 expression was sufficient to perturb Cu homeostasis, whole cell Cu content was measured by ICP-MS in the parental M21 and αV integrin-depleted M21L cells. As shown in Fig. 4A, the steady-state Cu level in the M21 cells was 0.24 ± 0.02 ng Cu/μg S whereas the M21L cells contained 0.62 ± 0.17 ng Cu/μg S (p = 0.019). Thus, the loss of αV integrin resulted in a 2.6-fold increase in intracellular Cu level. To provide further evidence that the loss of αV integrin influenced Cu homeostasis, the amount of exchangeable Cu was measured by staining live cells with coppersensor-3 (CS3). CS3 is a Cu chelator that readily penetrates cells and becomes fluorescent only when it binds Cu^+1^. Thus, it is capable of imaging the Cu^+1^ pools in living cells growing under basal conditions [28]. Similar to the changes in the basal Cu level detected by ICP-MS, CS3 staining indicated that the level of exchangeable Cu^+1^ was significantly increased in αV integrin-negative M21L cells when compared to the parental M21 cells (p = 0.016), and that restoration of αV expression returned the exchangeable Cu+1 to control levels (Fig. 4B and C).

**Figure 5:**
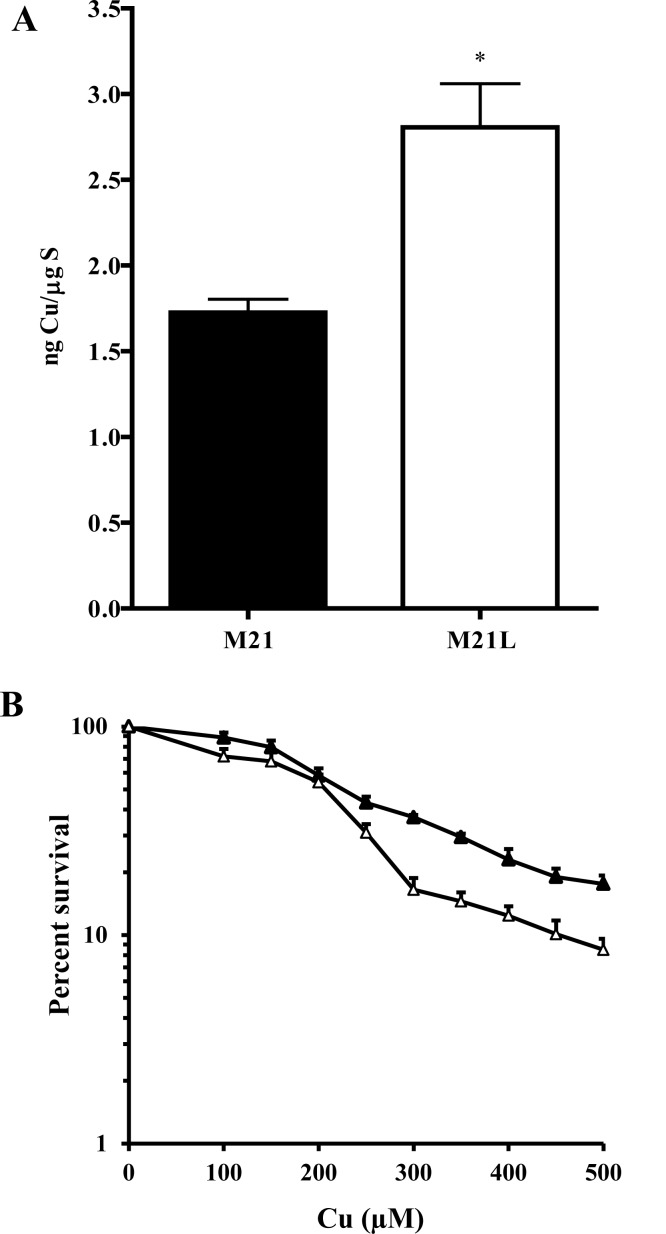
Effect of the loss of αV integrin on Cu accumulation and cytotoxicity (A) Total Cu accumulation following 24 h exposure to 100 μM Cu. (B) Inhibition of cell growth during 96 h continuous exposure of M21(▲) and M21L (Δ) cells to increasing concentrations of Cu. Vertical bars, ± SEM. *, p<0.05

We were curious as to whether the increased expression of CTR1 would permit enhanced accumulation of Cu when cells were exposed to concentrations of Cu that triggers internalization of CTR1 from the plasma membrane. Cellular Cu content was determined by ICP-MS following exposure of the cells to 100 μM Cu for 24 h. As shown in Fig. 5A, after this exposure the M21 and M21L cells contained 1.7 ± 0.14 and 2.8 ± 0.44 ng Cu/μg S, respectively (p = 0.041). The increase in both basal Cu content, and the level following exposure to a high level of Cu, is consistent with the changes observed in the expression of CTR1. To determine whether the differences in Cu accumulation translated into different tolerances to the cytotoxic effect of Cu, the growth rate of the M21 and M21L cells was measured during a 96 h exposure to increasing concentrations of Cu. As shown in Fig. 5B, although the difference in IC_50_ for the M21 and M21L did not reach statistical significance (IC_50_ 202.3 ± 23.5 versus 214.6 ± 16.7 μM, p = 0.078), an analysis of the slope of the overall curves in repeated experiments indicated a clear increase in sensitivity of the M21L cells to the higher Cu concentrations tested (p = 0.024). Thus, consistent with the higher expression of CTR1 and the greater accumulation of Cu, the M21L cells demonstrated increased susceptibility to the growth inhibitory effect of Cu.

### Loss of αV integrin increases Sp1 expression

**Figure 6:**
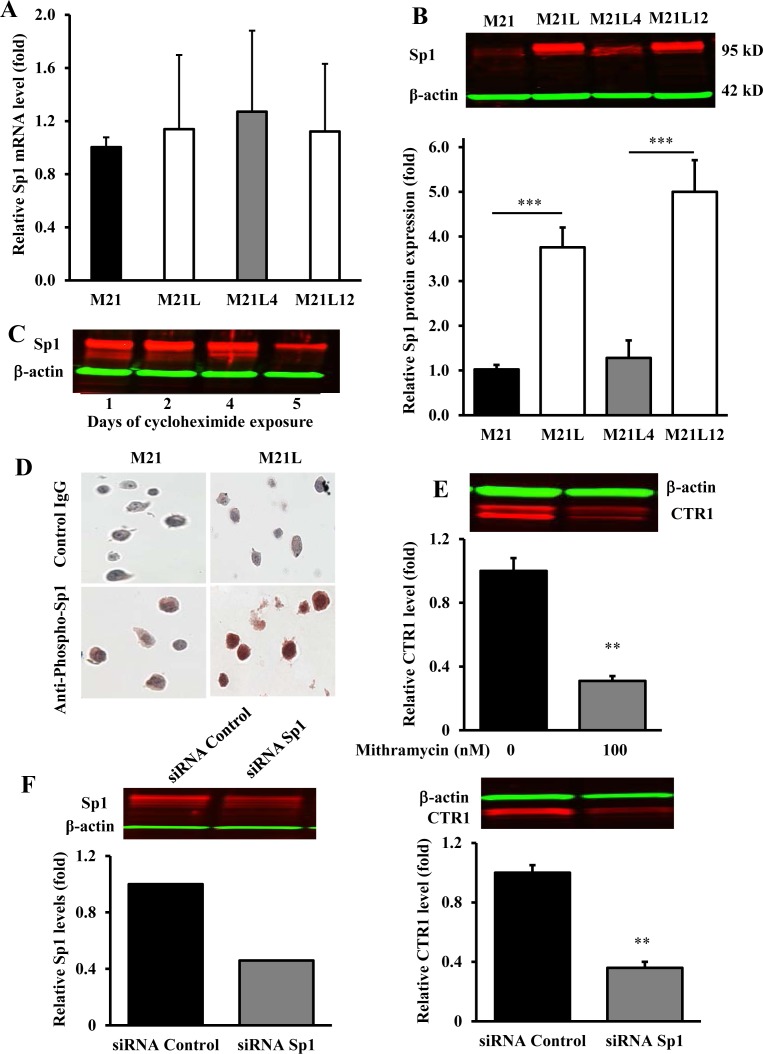
Loss of αV integrin enhances the expression of Sp1 (A) qRT-PCR analysis of the effect of the loss of αV integrin on mRNA expression of Sp1. (B) Representative Western blot showing the effect of αV integrin on Sp1 protein levels; the histogram shows the mean level of the protein determined from 3 independent experiments expressed as the fold change relative to that in the M21 cells after normalization to β-actin. (C) Western blot analysis of stability of Sp1 protein in M21 cells at the indicated times after the start of exposure to 30 μg/ml cycloheximide. (D) Immunohistochemical documentation of increased Sp1 phosphorylation on M21L cells; sections of paraffin-embedded cell pellets were stained with anti-phospho-Sp1 (pThr739) antibody. Magnification, ×200. (E) Down-regulation of CTR1 by Sp1 selective inhibitor mithramycin. M21 cells were treated with 100 nM for 24 h and examined for expression of CTR1 levels by Western blot analysis. (F) Reduction of Sp1 and CTR1 expression following RNAi-mediated knockdown of Sp1. Sp1 and CTR1 levels are expressed relative to that in the scrambled siRNA control. **p < 0.01; ***, p < 0.001. Vertical bars, ± SEM.

Sp1 is a ubiquitous transcription factor that has been shown to regulate CTR1 expression in mammalian cells [24]. To correlate Sp1 expression with CTR1 levels in the context of αV status, the steady-state Sp1 mRNA levels were measured in the M21, M21L, M21L4 and M21L12 cells. As shown in Fig. 6A, there was no significant difference in Sp1 mRNA expression across the four cell lines. In contrast, Sp1 protein level in the M21L cells was 3.76 ± 0.44- fold higher (p = 0.001) than that in M21L cells, and re-expression of αV integrin in the M21L4 cells significantly reduced Sp1 protein level as compared with the empty vector-transfected M21L12 cells (Fig. 6B). To determine whether Sp1 protein stability plays a major role in maintaining a high Sp1 level in the αV-negative cells, the stability of Sp1 protein was assessed by blocking new protein synthesis in M21 and M21L cells with cycloheximide and monitoring the disappearance of Sp1 for up to 5 d. Sp1 was found to be very stable with a half-life of > 4 d in M21 cells (Fig. 6C). An accurate determination of Sp1 half-life could not be made in M21L cells as they died after day 4 due to the toxicity of cycloheximide. As an alternative approach, the phosphorylation of Sp1 at T739 was quantified since this modification is known to enhance Sp1 protein stability [29]. Fig. 6D shows that a significant increase in the level of T739-phosphorylated Sp1 was found in the αV-depleted M21L cells compared with the parental M21 cells. These results suggest that the increase in Sp1 protein level that accompanied αV depletion is attributable to an increase in protein stability rather than a change in mRNA level. To further document a positive regulatory effect of Sp1 on CTR1 expression, endogenous Sp1 was suppressed by the Sp1 selective inhibitor mithramycin or knocked down by an siRNA targeted to Sp1. Treatment of M21 cells with 100 nM mithramycin for 24 h, which is known to be sufficient for inhibition of the transcription of Sp1- regulated genes [30], caused a significant reduction in CTR1 protein level (Fig. 6E). Similarly, siRNA-mediated inhibition of Sp1 in M21 cells, as confirmed by Western blot analysis (Fig. 6F), resulted in a significant decrease of CTR1 expression as compared with their scrambled siRNA transfected controls (Fig. 6F). This observation further attests to a pivotal role of Sp1 in the regulation of CTR1 expression and stabilization.

### Sequencing αV gene promoter region containing Sp1 binding sites

Our results are consistent with the concept that loss of αV expression is associated with increased expression of Sp1 and that Sp1 drives enhanced expression of CTR1 resulting in increased sensitivity to cDDP and Cu. Examination of the proximal region of the αV promoter sequence (-796/+207) (GenBank accession no. 23999) using MatInspector software disclosed 4 putative Sp1 binding sites: ^−610^GGCGGG, ^−537^CCCCGCCCCCGCCCC, ^−172^CCCCGCCC, ^−44^GGCGGG (Supplementary Fig. 1A). To explore the question of why the expression of αV was reduced in the M21L cells despite the very high level of Sp1, the portion of the αV promoter containing these sites (-796/+207) was sequenced. It was found that there were no mutations in the αV promoter region in either the M21 or M21L cells. Thus, the reduced expression of αV in the M21L cells cannot be attributed to mutational alteration of these potential Sp1 binding sites.

### Loss of αV integrin expression is not the result of DNA hypermethylation

Epigenetic changes that involve DNA methylation and alterations of chromatin structure can transcriptionally silence many genes. To determine whether the αV promoter is hypermethylated in the M21L cells they were treated with the demethylating agent 5-azacytidine and the change in expression of αV was quantified by flow cytometry using anti-αVβ3 antibody. As shown in Supplementary Fig. 1B, treatment with 5-azacytidine had no effect on αV expression in the M21L cells while it weakly induced αV expression in the M21 cells. This result suggests that the lack of αV expression in M21L cells is not attributable to a transcriptional block caused by the methylation of αV promoter in the M21L cells.

## DISCUSSION

CTR1 regulates the cellular pharmacology of cDDP and influences clinical responsiveness to cDDP treatment *in vivo* (reviewed in [21]). We have previously reported that loss of cell-cell interactions mediated by tight junctions results in cDDP resistance due to loss of CTR1 expression [31]. In the current study we sought to explore the effect of cell-matrix interactions mediated by integrins. Growth and survival of tumor cells are rigorously controlled by cell adhesion status, particularly the engagement of integrins and other ECM-binding surface receptors. Tumor cell interactions with ECM molecules lead to clustering of integrins and activation of intracellular signaling pathways through the focal adhesion kinase (FAK), integrin-linked kinase (ILK) and Src kinase [32]. The ability of integrins to regulate apoptosis is likely due to their capacity to activate the cell pro-survival signaling pathways further downstream of these cytoplasmic protein kinases such as phosphatidylinositol 3′-kinase (PI3K) and the serine /threonine kinase AKT, as well as the mitogen-activated protein kinase/extracellular regulated kinase (MAPK/ERK) [33]. Over the last decade since the discovery that ECM/integrin signaling provides a survival advantage to various cancer cell types, there has been an intensive effort to understand the mechanisms underlying the pro-survival function of integrins and how they modulate the sensitivity of cancer cells to chemotherapeutic agents. Early studies demonstrated that integrin-ECM interactions can protect small cell lung cancer [7], multiple myeloma [4] and glioma cell lines [8] from drug-induced apoptosis. Further studies identified integrin-mediated chemoresistance in other cancer cell types including various hematological malignancies, and demonstrated that it modulates sensitivity to several different classes of chemotherapeutic agents [34-37]. Adhesion-mediated chemo-resistance is often ascribed to integrin β-mediated stimulation of pro-survival signaling whereas understanding of the contribution of α-integrins is limited [33]. Since αV partners with 3 import β subunits, including β1, β3 and β5, we sought to determine how changes in expression αV modified sensitivity to cDDP and now provide evidence that it normally promotes survival of human M21 melanoma cells when they are insulted by exposure to this drug.

Selection of M21 cells for loss of αV expression resulted in a 2.8-fold increase in sensitivity to cDDP and this was associated with a 2.0-fold increase in the expression of CTR1 at the mRNA level and a 2.3-fold increase in the protein level. The specificity of this effect was documented by showing that re-expression of αV restored cDDP resistance and reduced CTR1 levels. That the increase in CTR1 expression associated with loss of αV was of functional significance and could account for the change in sensitivity was shown by the fact that it was accompanied by proportional increases in whole cell cDDP uptake and DNA adduct formation, and an increase in basal Cu levels and cytotoxicity all of which were reversed by re-expression of αV. There is now quite a large body of evidence, derived from multiple cell models, that CTR1 regulates the cytotoxicity of cDDP by affecting its uptake [38]. Elevated hCTR1 expression has now also been linked to favorable outcomes in both ovarian and lung cancer in which cDDP is used as part of primary therapy [39-41].

Further investigation disclosed that loss of αV was accompanied by an unusually large 3.8-fold increase in steady-state level of Sp1. That this was also a specific effect of αV loss was demonstrated by showing that re-expression of αV reversed the increase. No change in the level of Sp1 mRNA was detected by qRT-PCR suggesting that the altered level was the result of a change in protein stability. Attempts to determine the Sp1 half-life failed due to the fact that no reduction in Sp1 was detected prior to the time when the cells became overtly sick due to the toxicity of the cycloheximide. However, that Sp1 was indeed stabilized by αV loss was suggested by the finding that phosphorylation at T739 was increased in αV-negative M21L cells. The observation that the *hCTR1* promoter reporter detected 1.8-fold greater activity in the M21L cells is consistent with the conclusion that the increase of CTR1 expression was a direct result of the increased level of Sp1 especially since the reporter activity mirrored the Sp1 level in all 4 cell lines. Sp1 belongs to the specificity protein/Krüppel-like factor family of transcription factors that bind to the GC-rich promoter element through three Cys_2_His_2_-type zinc-fingers. It is ubiquitously expressed in many tissues and regulates a wide variety of cellular processes [42]. Interestingly, the αV promoter contains 4 predicted Sp1 binding sites, and Sp1 has been reported to increase αV expression once bound to the promoter [43]. Given that Sp1 controls the expression of many different genes it seems unlikely that an abnormality of just the αV promoter, if it exists, would trigger such a large compensatory increase in Sp1 expression. In addition, we did not detect any mutations in the Sp1 binding sites in the αV promoter of the M21L cells. Epigenetic mechanisms have been linked with the inactivation of the promoters of integrin family members [44]. In an effort to determine whether the loss of αV integrin expression in the M21L cells was due to DNA methylation we treated the cells with the demethylating agent 5-azacytidine but found that this did not restore αV expression. The basis for the lack of αV expression in the M21L cells, and the mechanism by which this increases Sp1 levels remains an open question.

While most studies of the mechanisms by which integrins modulate drug sensitivity have focused on changes in the expression of proteins that control apoptosis, the findings of the current study provide compelling evidence that αV can modulate drug sensitivity through an effect on a plasma membrane transporter. While loss of tight junctions and adherens junctions that mediate cell-cell interactions is associated with reduced expression of CTR1 and cDDP resistance [31], the loss of cell-ECM interaction that presumably accompanies loss of αV expression is associated with an increase in CTR1 and enhanced cDDP sensitivity. Since even quite small changes in cDDP sensitivity measured *in vitro* translate to large differences in cDDP efficacy *in vivo* [45-47], further investigation of pharmacologic modulation of αV expression or function is of substantial interest.

## METHODS

### Cells and cell culture

Human melanoma cell line M21, M21 variants M21L (α_v_ negative), M21L4 (α_v_ positive), and M21L12 (α_v_-negative transfection control) were described previously [22, 23]. ATCC authentication was performed using short tandem repeat DNA profiling. These cells were cultured in RPMI 1640 medium containing 10% fetal calf serum, 2 mM L-glutamine, 100 U/ml penicillin and 100 mg/ml streptomycin.

### Drugs and reagents

A clinical formulation of cDDP was obtained from the UC San Diego Moores Cancer Center pharmacy. Cupric sulfate was obtained from Sigma (St. Louis, Mo.). The drugs were diluted to the desired concentrations in RPMI medium (Thermo Scientific, Logan, UT). The Detergent Compatible Protein kit was purchased from BioRad (Hercules, CA) and the tetrazolium compound WST-8 (Cell Counting Kit-8, CCK-8) from Dojindo Molecular Technologies (Rockville, MD). The demethylating agent 5-azacytidine (AZA) was purchased from Sigma. Mithramycin was purchased from Sigma.

### Flow cytometry

Cell surface expression of αVβ3, αVβ5 and β1 integrins was determined by flow cytometric analysis. Briefly, single cell suspension containing 5 × 10^5^ cells in 2% bovine serum albumin (BSA) PBS was stained on ice for 45 min using anti-αVβ3, anti-β1, anti-αVβ5 (provided by Dr. Dwayne Stupack, University of California-San Diego) or isotype negative control (BD Biosciences) antibodies. After washing with 2% BSA-containing PBS, cells were incubated with Alexa Fluor 488-conjugated goat anti-mouse F(ab')2 fragments (Invitrogen, Carlsbad, CA). After three subsequent washing steps, 1 × 10^4^ cells were assessed for cell surface expression of the integrins using a FACScan flow cytometer (BD Biosciences, San Jose, CA).

### Cell survival assay

Cells were plated into 96-well plates at a density of 3,000 cells per well and allowed to adhere overnight. The cells were then exposed to increasing concentrations of cDDP. After 96 h, the effect of cDDP on the cell survival was determined using the tetrazolium compound WST-8 assay as described previously [48]. All experiments were repeated at least three times using three cultures for each drug concentration.

### qRT-PCR

For quantification of CTR1 mRNA levels by qRT-PCR, cDNA was generated from mRNA isolated using TRIzol (Invitrogen, Carlsbad, CA). Purified mRNA was converted to cDNA using oligo(dT)20 primer and the SuperScript III First-Strand kit (Invitrogen, Carlsbad, CA). qRT-PCR was performed on an MyIQ qPCR machine (Bio-Rad Laboratories, Hercules, CA). The forward and reverse primers for CTR1 and glyceraldehyde-3- phosphate dehydrogenase (GAPDH) were, respectively, actgttgggcaacagatgct and ctgctgctactgcaatgcag, tcaccaccatggagaaggc and gctaagcagttggtggtgca. Reactions were prepared using iQ SYBR Green Supermix (Bio-Rad Laboratories, Hercules, CA) according to the manufacturer's recommendations. Samples were prepared in quadruplicate with 3 independent sample sets being analyzed. Analysis was done using the Bio-Rad iQ5 system software.

### Measurement of Pt and Cu accumulation

Whole cell Pt and Cu content was measured as previously reported [49]. All data presented are the means of at least 3 independent experiments, each performed with 6 cultures per concentration tested. For measurement of Pt in DNA, cells were lysed and DNA harvested using DNAzol (Invitrogen, Carlsbad, CA) according to the manufacturer's protocol. For normalization, DNA was measured prior to addition of nitric acid using a Nanodrop 2000 spectrophotometer (Thermo Scientific, Wilmington, DE). The DNA samples were then digested in nitric acid and prepared prior to measurement of Pt by ICP-MS as previously described [49].

### Molecular imaging of endogenous basal Cu with copper sensor-3 (CS3)

Exchangeable Cu within live cells was measured with CS3 as previously described [28] with minor modifications. Cells were loaded with a mixture of 8-[N,N-Bis(3′,6′-dithiaoctyl)-minomethyl]-2,6-diethyl-4,4-dimethoxy-1,3,5,7-tetramethyl-4-bora-3a,4a-diaza-s-indacene (Copper sensor-3, CS3) at 2 μM and Hoechst 33342 (5 μM in RPMI 1640 at 37 °C for 10 min, washed and imaged in fresh RPMI 1640, and excited at 543 nm with a HeNe laser for CS3 and at 405 nm with a diode laser for Hoechst 33342. Confocal fluorescence imaging was performed at the University of California San Diego Cancer Center Digital Imaging Shared Resource using a Zeiss LSM510 confocal microscope system (Carl Zeiss, Inc., Thornwood, NY). After staining, 3 randomly chosen fields were imaged from each culture using 32 Z-slices with a step size of 0.8 μm in Z direction. Image J software was then used in the average intensity projection mode to exclude the Hoechst 33342 fluorescence and determine the mean CS3 fluorescence in an area of interest drawn around each of 10 cells from each of the 3 fields in a given culture. The mean fluorescence in the CS3 channel per unit area from 4 areas of interest drawn on background parts of the image containing no cells was subtracted from this value. Thus, from each culture a mean value was obtained for the total of 30 cells analyzed. Each experiment was repeated 3 times on separate days, and the mean values for each type of culture were averaged. Thus, the data was reported as the mean ± SEM (N = 3) of the average obtained from 30 cells in each of 3 repeats. A two-tailed Student's *t* test was used to establish statistical significance.

### Preparation of post-nuclear membrane pellets

Total cell membranes were isolated by the method of Tischkau *et al*.[50] with slight modifications. Briefly, cells were homogenized in ice-cold homogenizing buffer (0.25 M sucrose, 10 mM Tris-HCl pH 7.4, 2 mM EDTA, protease/phosphatase inhibitors). Homogenates were centrifuged at 1000 × g for 10 min at 4°C to remove pelleted nuclear fraction. Supernatants were centrifuged at 135,000 × g for 30 min to yield crude cytosol and crude membrane pellet (P_2_). The membrane pellet was solubilized in the homogenizing buffer for a minimum of 1 h at 4 °C and the protein concentration was measured by the DC protein assay.

### Luciferase assay

Cells were co-transfected with the hCTR1 promoter-firefly luciferase reporter construct pGL3-hCTR1(-227) [24] and a β-galactosidase-containing vector pCMXβgal by using the FuGENE transfection reagent (Roche Diagnostics) according to the manufacturer's instructions. Twenty-four h after transfection, cells were washed with PBS and lysed in a lysis buffer (Promega, Madison, WI), and light emission was detected in the luciferase reporter assay system (Promega) using a microtiter plate luminometer (MicroBeta TriLux, Gaithersburg, MD). The luciferase activities were normalized for variations in transfection efficiency by using the β-galactosidase as an internal control, and were expressed as fold induction relative to the control cultures. Experiments were repeated 3 times with duplicate cultures.

### Western blot analysis

The protein from the solubilized post-nuclear membrane pellet was loaded on SDS-PAGE and separated by electrophoresis. A Bio-Rad Trans-Blot system was used to transfer the proteins to Immobilon-P FL membranes (Millipore, Bedford, MA). Membranes were blocked for 1 h at room temperature in the Odyssey Blocking Buffer (Li-Cor; Lincoln, NE), followed by incubation overnight at 4oC with rabbit monoclonal anti-CTR1 antibody (Epitomics; Burlingame, CA) at 1:1000 dilution and with a mouse monoclonal anti-human transferrin receptor (Invitrogen; Carlsbad, CA) diluted 1:2000 by the Odyssey Blocking Buffer containing 0.1% Tween 20. After washing 4 times for 5 min each at room temperature in PBS containing 0.1% Tween 20 the blots were incubated for 1 h at room temperature with fluorescently labeled secondary antibody (Li-Cor; Lincoln, NE) diluted 1:10,000 in the Odyssey Blocking Buffer containing 0.1% Tween 20 and 0.02% SDS. After 4 washes for 5 min each in 0.1% Tween 20 PBS and rinse with PBS the blots probed with fluorescently labeled antibody were imaged using an Odyssey Infrared Imager (Li-Cor; Lincoln, NE). To examine the effect of suspension growth on CTR1, αV integrin and E-cadherin expression, adherent M21 cells were cultured in ultra-low attachment 6-well plates (Corning Incorporated, Corning, NY) overnight and the whole cell lysates were harvested for Western blot analysis using the anti-CTR1, anti-aV (Cell Signaling Technology, Danvers, MA) and anti-E-cadherin (Santa Cruz Biotechnology, Santa Cruz, CA) or anti-β-actin antibody (Sigma-Aldrich, St. Louis, MO).

### Assessment of Sp1 stability

M21 and M21L cells were incubated with 30 μg/ ml cycloheximide for 1-5 d, washed 3 times with PBS, and lysates were harvested for Western blot analysis using anti-Sp1 antibody (sc-14027, Santa Cruz Biotechnology). Bands were quantified by using an Odyssey infrared imager (Li-Cor Biosciences), and fold changes were normalized to β-actin levels.

### RNA interference

Sp1-specific siRNA mix (SignalSilence® SP1 siRNA II #12106) and a control non-targeting siRNA (Control siRNA: sc-37007) was obtained from Cell Signaling and Santa Cruz Biotechnology, respectively. Transfections were performed using the Lipofectamine 2000 transfection reagent according to the manufacturer's instructions.

### Statistical analysis

All two-group comparisons utilized Student's *t*-test with the assumption of unequal variance. Data are presented as mean ± SEM.

## SUPPLEMENTARY FIGURE


