# Supplementary material for: Integrin αV modulates the cellular pharmacology of copper and cisplatin by regulating expression of the influx transporter CTR1
Source: Oncoscience. 2014 Mar 24;1(3):185–95. doi: 10.18632/oncoscience.22 (PMC4278295; doi:10.18632/oncoscience.22)
Supplement: Supplementary file 1 [file oncoscience-01-0185-s001.pdf]

Integrin αV modulates the cellular pharmacology of copper and cisplatin by regulating expression of the influx transporter CTR1 – Lin et al

A

-800 GCAAGAGGCTATGCTGGCTTTCTGGAAATCCGTAATTAAGTTCAGTGTTTGTGGAATGGAGTGTAATTAACGACCATTAATTAACAGGGTTTCGTT

-700 CGTGAGAGCCTGCCAGGCTAAGCAGCAGAAACACGAGAAACTGATAAAAAGCTTTCCTCATTTTTAAACAACAGTCGCACGGAAGTTCCCGGCGGGACAA

-600 GGGAACGTGGGTGCCCTTGCTACTCCCGTGGACGCGGGTAGATTGGGACGCTGGACCGTATCTCCCCGCCCCCGCCCCCACGCCTCCTCAGGTGCTCAGC

-500 CTGAGGCCTTCGTCCAGGAGCGCTGCCGCTGACCCAGGCTCAGGAGCTGGGGGCCCCTGCACAGACGCCCAGGTCTCGGGACAGGCGGCGACTGCACTCA

-400 CGGAAGTACGCTGAGCTCTCCCCTGTAGAAGGGCGCCTCTCCTCCCCCACTTCCTCCTCCAGCTCCACAGCAGCCTCCCGGGCCGGCTCCTCCTCCTTCC

-300 AGGTCTCCTCCCAGTGCCGCCGCGGCTCTCAGGCCTGAGGTGCGGCGCTCACCCCGGCAGTCCCCAGCCTCAGACGCTGCGTGGAGCGGCGGAGCCGGAG

-200 GGAAGCAAAGGACCGTCTGCGCTGCTGTCCCCGCCCCGCGCGCTCTGCGCCCCTCGTCCCTGGCGGTCGCTCCGAAGCTCAGCCCTCTTGCCTGCCCCGG

-100 AGCTGTCCCGGGCTAGCCGAGAAGAGAGCGGCCGGCAAGTTTGGGCGCGCGCAGGCGGCGGGCCGCGGGCACTGGGCGCCTCGCTGGGGCGGGGGGAGGT

+1 GGCTACCGCTCCCGGCTTGGCGTCCCGCGCGCACTTCGGCGATGGCTTTTCCGCCGCGGCGACGGCTGCGCCTCGGTCCCCGCGGCCTCCCGCTTCTTCT

+101 CTCGGGACTCCTGCTACCTCTGTGCCGCGCCTTCAACCTAGACGTGGACAGTCCTGCCGAGTACTCTGGCCCCGAGGGAAGTTACTTCGGCTTCGCCGTG

+201 GATTTCT

B

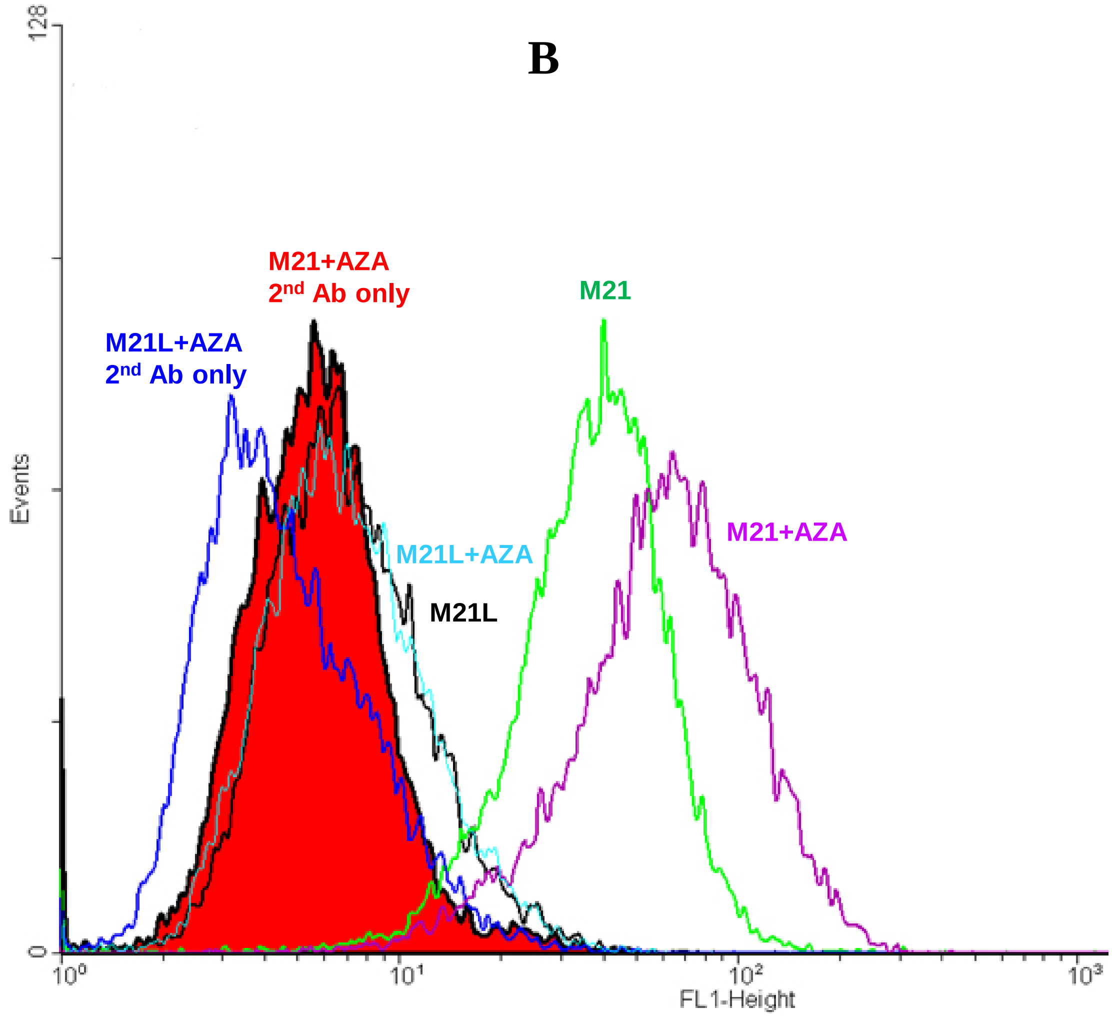

Supplementary Figure 1: Putative Sp1 binding sites in the αV promoter and effect of 5-aza-cytidine on αV expression. A, map of putative Sp1 binding sites in the αV promoter. B, effect of treatment of M21 and M21L cells with 2 μM 5-azacytidine for 4 d on αV expression determined by flow cytometric analysis using anti-αVβ3 antibody. The controls include the untreated cells followed by staining with both primary and FITC-conjugated secondary antibodies, and the 5-aza-cytidine-treated cells stained with secondary antibody only.
